# DNA strand breaks and TDP-43 mislocation are absent in the murine *hSOD1^G93A^* model of amyotrophic lateral sclerosis *in vivo* and *in vitro*

**DOI:** 10.1371/journal.pone.0183684

**Published:** 2017-08-23

**Authors:** Diane Penndorf, Vedrana Tadić, Otto W. Witte, Julian Grosskreutz, Alexandra Kretz

**Affiliations:** Hans Berger Department of Neurology, Jena University Hospital, Jena, Thuringia, Germany; International Centre for Genetic Engineering and Biotechnology, ITALY

## Abstract

Mutations in the human *Cu/Zn superoxide dismutase type-1* (*hSOD1*) gene are common in familial amyotrophic lateral sclerosis (fALS). The pathophysiology has been linked to, e.g., organelle dysfunction, RNA metabolism and oxidative DNA damage conferred by SOD1 malfunction. However, apart from metabolically evoked DNA oxidation, it is unclear whether severe genotoxicity including DNA single-strand breaks (SSBs) and double-strand breaks (DSBs), originates from loss of function of nuclear SOD1 enzyme. Factors that endogenously interfere with DNA integrity and repair complexes in *hSOD1*-mediated fALS remain similarly unexplored. In this regard, uncontrolled activation of transposable elements (TEs) might contribute to DNA disintegration and neurodegeneration. The aim of this study was to elucidate the role of the fALS-causing *hSOD1*^*G93A*^ mutation in the generation of severe DNA damage beyond well-characterized DNA base oxidation. Therefore, DNA damage was assessed in spinal tissue of *hSOD1*^*G93A*^-overexpressing mice and in corresponding motor neuron-enriched cell cultures *in vitro*. Overexpression of the *hSOD1*^*G93A*^ locus did not change the threshold for severe DNA damage *per se*. We found that levels of SSBs and DSBs were unaltered between *hSOD1*^*G93A*^ and control conditions, as demonstrated in post-mitotic motor neurons and in astrocytes susceptible to replication-dependent DNA breakage. Analogously, parameters indicative of DNA damage response processes were not activated *in vivo* or *in vitro*. Evidence for a mutation-related elevation in TE activation was not detected, in accordance with the absence of TAR DNA binding protein 43 (TDP-43) proteinopathy in terms of cytoplasmic mislocation or nuclear loss, as nuclear TDP-43 is supposed to silence TEs physiologically. Conclusively, the superoxide dismutase function of SOD1 might not be required to preserve DNA integrity in motor neurons, at least when the function of TDP-43 is unaltered. Our data establish a foundation for further investigations addressing functional TDP-43 interaction with ALS-relevant genetic mutations.

## Introduction

Amyotrophic lateral sclerosis (ALS) represents the most frequent lethal disorder of the motor system and is characterized by a relentless and progressive loss of upper and lower motor neurons (MNs) of the motor cortex, brain stem and MNs of the spinal cord. The provenance is complex and yet untargeted by efficient therapeutic strategies. Clinically, devastating symptoms manifest with progressive spastic and atrophic paralysis, impairments in speech and swallowing and finally lead to respiratory failure 3–5 years after the onset [1]. Among the approximately 20 causative gene loci and about 30 disease-modifying genetic factors identified to date [2], numerous mutations in the human *Cu/Zn superoxide dismutase 1* (*hSOD1*) gene are accounting for approximately 20% of familial ALS (fALS) and 1–2% of sporadic ALS (sALS) cases [1, 2]. *SOD1* mutations involving all five exons are predominantly single-base-pair substitutions, which are commonly linked to an aggressive course with rapid clinical deterioration, including those variants implicating a p.G93A mutation [2]. Though disease pathogenesis generally implicates cellular, molecular and metabolic alterations, the underlying etiology is still debated. Likewise, the role of severe DNA damage and endogenous DNA repair strategies in ALS initiation and progression are ill-defined. This topic implies a notable role for ventral horn spinal MNs, the neuronal population that is most afflicted in the disease pathology, which must cope with numerous DNA damaging agents, such as oxidizing molecules, in cases of both fALS and sALS [3, 4]. Reactive oxygen species (ROS) are among the most crucial noxious metabolites impacting MNs due to their immense energetic requirements and metabolic turnover. Intracellular ROS concentrations are increased in *hSOD1*-dependent fALS and may contribute considerably to genomic instability [5, 6]. ROS preferentially target DNA bases or sugar residues, resulting in DNA single-strand breaks (SSBs). They can also induce the formation of DNA double-strand breaks (DSBs), e.g., if the transcription apparatus is affected at locations where ROS-mediated lesions eventuate in a narrow sequence.

Accounting for the toxic gain of function assumed to originate from mutant *hSOD1* together with a loss of function of naïve SOD1 enzyme in the nucleus [7], clinical severity might thus arise from oxidative stress-induced severe DNA damage. This may include DNA SSBs and DSBs, in particular if they fail to become repaired, or remain mis-repaired. In accordance with this notion, the levels of 8-hydroxy-deoxyguanosine, a marker of oxidative DNA demise, are increased in spinal cord of sALS and fALS patients [8]. Likewise, DNA damage is rising during ROS exposure in cultured NSC34 cells carrying a *hSOD1*^*G93A*^ mutation compared with wild type transfectants [7]. However, whether this mutation causes a predisposition to DNA strand breaks under ALS-like conditions *in vivo*, apart from artificially high ROS levels, has remained unexplored.

Aside from ROS-induced DNA damage, the role of endogenously generated DNA demise in *hSOD1*-mediated fALS, in particular in non-dividing MNs lacking replication stress as a considerable physiological source of DNA breakage has been similarly uninvestigated. Uncontrolled activation of transposable elements (TEs) and their progressive de-repression are thus discussed as critically involved in DNA damage and central nervous system (CNS) neurodegeneration [9–11]. There are indications of a contribution of certain TEs to the pathology of ALS, at least in sporadic cases [12]. However, whether TE activation is implicated in *hSOD1*-related MN degeneration has remained an open question. TEs represent repetitive DNA elements that account for approximately 30%-50% of the mammalian genome [13]. They are actively mobile within the DNA strand and exhibit the highest activation in neuronal tissue, for reasons that still have to be defined [14, 15]. Genomic TE integration is capable of disturbing gene function and causing DNA strand breaks [16] if not properly prohibited, e.g., by TE DNA methylation, histone modifications and post-transcriptional silencing [9]. Regarding the latter, there is evidence that nuclear transactive response DNA-binding protein 43 (TDP-43) targets TE-derived transcripts and thus plays a physiological role in silencing TE transposition [10]. Pathological alterations, e.g., nuclear-to-cytoplasmic translocations, of the RNA/DNA-binding protein TDP-43 are a well-accepted hallmark of both fALS and sALS [17]. However, as one exceptional entity, *hSOD1*-related fALS is assumed to be devoid of cytoplasmic TDP-43 aggregation [17]. In the absence of a cytoplasmic proteinopathy, a nuclear loss of TDP-43 might critically impair its suppressor function on the activation of TE moieties. To date, it is unknown whether TDP-43 stabilization of DNA integrity is altered in MNs carrying a *hSOD1* mutation in spite of the putative absence of a TDP-43 mislocation or whether the extent of the *hSOD1* mutation impacts on genomic integrity in general.

Hence, the aim of this study was to elucidate the role of the rapidly progressive fALS-causing p.G93A *hSOD1* mutation in the generation of severe DNA damage. To allow an in-depth evaluation beyond well-characterized DNA base oxidation, e.g., caused by ROS overload, DNA SSB and DSB events were examined in the *hSOD1*^*G93A*^ murine model *in vivo* as a function of the disease stage, as well as in MNs *in vitro*. Apart from overall DNA damage, the occurrence of DNA breakage was specified for non-replicative MNs and dividing astrocytes in MN-enriched cell cultures with respect to the *hSOD1*^*G93A*^ mutation. Moreover, the DNA integrity and DNA damage response (DDR) were assessed in light of the DNA-stabilizing function of nuclear TDP-43, mediated by its interaction with TE transcripts, particularly in spinal MNs.

Despite its well-established toxic gain of function on SOD1 enzyme activity, our results indicate that the p.G93A mutation at the *hSOD1* locus does not change the threshold for severe DNA SSBs or DSBs *per se*, at least when assessed in a murine context. This was demonstrated in post-mitotic MNs and in astrocytes susceptible to replication-dependent DNA breakage. Analogously, parameters indicative of DDR processes were not activated *in vivo* or *in vitro*. The absence of TDP-43 proteinopathy in terms of cytoplasmic mislocation or nuclear loss in the *hSOD1*^*G93A*^ model, irrespective of the disease stage, might underscore the role of TDP-43 in DNA stabilization. Consistent with the lack of TDP-43 decompartmentalization and nuclear loss, we did not obtain evidence of elevated TE activation during the severe disease stage.

We conclude that the superoxide dismutase function of SOD1 might not be required to preserve DNA integrity in MNs, at least when the function of TDP-43 is unaltered. Our data might establish the foundation for further investigations addressing the interaction of the DNA stabilizing function of TDP-43 with ALS-relevant genetic mutations.

## Materials and methods

### Mice

Transgenic mice overexpressing mutant *hSOD1*^*G93A*^ (B6.Cg-Tg(SOD1*G93A)1Gur/J; Jackson Laboratory, Bar Harbor, ME, USA; stock number 004435) were used to mimic ALS-like conditions [18] prior to and after the onset of disease-related symptoms. The disease stage was assessed by age in parallel with a clinical score as detailed in S1 Table including the definition of humane endpoints. Clinical state was examined at least weekly. As assessed from a three-years characterization of approximately 200 male *hSOD1*^*G93A*^ mice in our facility, the onset of disease manifestation by hind limb paresis occurred at an average age of 18.8 ± 0.1 weeks. In this study, exclusively male animals were included and designated as ‘diseased’ as soon as a clinical score of 1–2 was reached, usually occurring at an age of 19–23 weeks. Presymptomatic mice were phenotypically normal and were included at 8–9 weeks of age. Since they were bred on an identical background, 11-13-week-old male C57BL/6J mice served as controls. For *in vitro* experiments, B6.Cg-Tg(SOD1*G93A)1Gur/J transgenic and non-transgenic embryos at E13 were used as cell donors. Genotyping was performed by genomic PCR immediately before cell plating. Conditions of cell replication and impaired DNA repair were gained in brain tissue from adult NOD.CB17-Prkdcscid/NCrHsd mice displaying defective DDR responses and repair of DSBs, with or without fronto-cortical xenografting of highly proliferative LN229 human glioblastoma cells (kindly provided by J. Walter, Department of Neurosurgeries, Jena University Hospital). Mice were housed and mated under standardized, pathogen-free conditions with unlimited access to standard diet and water and exposed to a 14/10-hours light/dark cycle. All animal interventions were conducted in compliance with the Directives of the Protection of Animals Act and were approved by the animal welfare authorities of Thuringia (accreditation number: 02-046/14).

### Comet assay

Adult mice deeply anesthetized with vaporized Isoflurane (Isolfurane CP^®^, CP Pharma, Burgdorf, NI, Germany) in a specialized chamber were transcardially perfused with ice-cold PBS and decapitated. To prepare single cell suspensions, the cervical and thoracic spinal cords were micro-dissected and incubated in calcium-free Hibernate^®^ A (BrainBits LLC, Springfield, IL, USA) supplemented with 0.5 mM GlutaMAX^™^ (Gibco^™^/Thermo Fisher Scientific, Waltham, ME, USA), 0.132 M D-(+)-trehalose dihydrate (Sigma-Aldrich, Munich, BY, Germany), 310 U/ml DNase I (Sigma-Aldrich) and 1 mg/ml collagenase type IA (Sigma-Aldrich) for 30 min at 37°C under gentle agitation. The working solution was then replaced with a solution containing 2 mg/ml papain (Worthington, Lakewood, NJ, USA) instead of collagenase. Cell disseminates were maintained at 37°C for 30 min under repeated trituration using a fire-polished Pasteur pipette to obtain a single cell suspension. Enzymatic digestion with papain was finally blocked by the addition of ice-cold 0.1% ovomucoid (Sigma-Aldrich) in Hibernate^®^ A working solution, in which the enzymes were replaced with 0.1% BSA (Serva, Heidelberg, BW, Germany). This suspension was sequentially passed through Falcon^™^ cell strainers with a mesh size of 100 μm and 40 μm (Thermo Fisher Scientific, Waltham, ME, USA) before being centrifuged at 500 x *g* for 5 min. As a positive control, cortical cells from the NOD.CB17-Prkdcscid/NCrHsd strain devoid of LN229 xenografts were used. The cell pellet was resuspended in ice-cold PBS and diluted to a final concentration of 100,000 cells/ml. The comet assay was performed using the OxiSelect^™^ Comet Assay Kit (3-well slides; Cell Biolabs, San Diego, CA, USA), and all steps were performed according to the manufacturer’s manual. Briefly, the diluted single cell suspension was mixed at a 1:10 ratio with OxiSelect^™^ Comet agarose, plated on pre-coated slides and incubated for 15 min at 4°C. All steps prior to single cell gel electrophoresis were performed in the dark to avoid additional UV light-induced DNA damage. For cell lysis and DNA unwinding, the slides were transferred to 4°C conditions, incubated in lysis buffer for 50 min and then immersed in alkaline solution for 30 min. To detect both DNA SSBs and DSBs, single cell gel electrophoresis was performed under alkaline conditions (15 V, 300 mA, 30 min). The slides were rinsed in water, dehydrated in 70% ethanol for 5 min and then stained with 1x Vista Green DNA-dye solution for 15 min. Random images of at least 60 comets out of approximately 2,250 cells distributed among 3 separated wells per animal were obtained using an Axioplan2 Imaging microscope (20x air objective; Zeiss, Oberkochen, BW, Germany) coupled to an AxioCam HRc camera (Zeiss). For semi-automated comet analysis, the CASP software was employed [19]. For comet scoring, the features ‘Percent of Tail DNA’ (% TailDNA), which represents the relative tail intensity, and the ‘Tail Moment’ were selected as comparative parameters. The ‘Tail Moment’ provides information on the length of the comet tail, which is multiplied with the % TailDNA. Although the ‘Tail Moment’ is frequently used, the application of % TailDNA bears the advantage that it is linearly correlated with the break frequency and allows for a visual comet scoring gradation as described by Collins (2004) [20]. Based on this visual comet scoring algorithm, comets were classified by their % TailDNA according to the following categories: category 0, 0–20% TailDNA; category 1, 20–40% TailDNA; category 2, 40–60% TailDNA; category 3, 60–80% TailDNA; and category 4, 80–100% TailDNA. In total, comets from 3 animals per group were investigated.

### Motor neuron-enriched cell culture

Pregnant mice were sacrificed by cervical dislocation with subsequent decapitation. MNs derived from spinal cords of E13 mouse embryos were cultured on an astrocytic feeder layer as previously described [21, 22]. Briefly, spinal cords of transgenic and non-transgenic embryos were dissected, cut into pieces and pooled in HBSS (Gibco^™^/Thermo Fisher Scientific) containing 1% penicillin-streptomycin (Gibco^™^/Thermo Fisher Scientific) and 1 M HEPES (Roth, Karlsruhe, BW, Germany), according to the genotype. The tissue was digested in 0.1% trypsin in HBSS for 15 min at 37°C and triturated with a fire-polished Pasteur pipette subsequent to DNase (AppliChem, Darmstadt, HE, Germany) treatment to obtain a single cell solution. The MNs and glial cells were separated by centrifugation in a 6.2% OptiPrep density gradient solution (Axis-Shield/Alere Technologies AS, Oslo, Norway) diluted in L-15 medium (Sigma-Aldrich). Separated astrocytes (50,000 cells/coverslip) were seeded on poly-D-lysine hydrobromide (Sigma-Aldrich)-coated 12-mm coverslips (Marienfeld GmbH & Co. KG, Lauda-Königshofen, BW, Germany) to create a feeder monolayer. During the first week, the astrocytic feeder layer medium was composed of L-glutamine-free DMEM/Ham’s F-12 medium (Gibco^™^/Thermo Fisher Scientific) at a 1:1 ratio supplemented with 1% penicillin-streptomycin and 10% fetal calf serum (PAN Biotech, Aidenbach, BY, Germany). The fetal calf serum was then replaced with equal amounts of horse serum. After confluence was reached, astrocytic cell division was blocked by addition of 5 μM 1-β-D-Arabinofuranosylcytosine (Calbiochem^®^/Merck Chemicals, Darmstadt, HE, Germany), which was removed by washing after 24 h. MNs (15,000–30,000 cells/coverslip) were seeded on the prepared feeder monolayer of the matching genotype. MNs were grown for 14 DIV in L-glutamine-free Neurobasal medium (Gibco^™^/Thermo Fisher Scientific) supplemented with 2% 50x B27^®^ Supplement (Gibco^™^/Thermo Fisher Scientific), 0.2% 100x N-2 Supplement (Gibco^™^/Thermo Fisher Scientific), 1 mM L-glutamine (Gibco^™^/Thermo Fisher Scientific), 2% horse serum (Gibco^™^/Thermo Fisher Scientific), 1% penicillin-streptomycin and 2 ng/ml human recombinant BDNF (PeproTech, Rocky Hill, NJ, USA). For all culturing steps, the 5%-CO_2_-humidified incubator was set at 37°C.

### Immunofluorescence

Adult mice were deeply anesthetized with vaporized Isoflurane in a specialized chamber, transcardially perfused with ice-cold PBS and with freshly prepared 0.1 M sodium cacodylate trihydrate (Sigma-Aldrich) buffer containing 4% PFA (pH 7.3) and subsequently decapitated. The cervical spinal cords were micro-dissected and kept overnight in the perfusion solution at 4°C for post-fixation. They were then cryoprotected in 15% sucrose (Roth) for 48 h at 4°C and frozen in 2-methylbutane. Tissue slices were cut (25 μm for cervical spinal cord; 16 μm for brain) with a cryotome (Leica Biosystems, Wetzlar, HE, Germany), dried at 37°C and subjected to an antigen retrieval step by incubating the tissue slices in 10 mM sodium citrate dihydrate (Sigma-Aldrich) buffer (pH 9.0) at 80°C for 30 min or incubating the MN-enriched cultures in 1% SDS (Serva) in PBS for 10 min subsequent to cell fixation in 4% PFA/PBS for 20 min. Non-specific epitope binding was blocked by incubation in 10% NDS (Merck Chemicals) or NGS (Gibco^™^/Thermo Fisher Scientific) in 3% BSA/PBS containing permeabilizing 0.3% Triton X-100 for at least 2 h.

Primary antibodies comprised mouse anti-SMI32 (1:500 in tissue, 1:1,000 in MN culture; monoclonal; Covance, Princeton, NJ, USA, RRID: AB_2564642) as a MN marker and rabbit anti-βIII-tubulin (1:200 in tissue, 1:250 in MN culture; polyclonal; Sigma-Aldrich, RRID: AB_262133) as a general neuronal marker. A polyclonal rabbit anti-TDP-43 was used *in vivo* and *in vitro* (1:1,000 in tissue, 1:100 in MN culture; polyclonal; Acris Antibodies, Herford, NW, Germany, RRID: AB_615042). Rabbit anti-53BP1 (1:1,000 in tissue, 1:8,000 in MN culture; polyclonal; Abcam, Cambridge, ENG, UK, RRID: AB_722497) and rabbit anti-γH2AX (1:500 in MN culture; polyclonal; Abcam, RRID: AB_303388) were taken as indicators of DSBs. Mouse anti-PCNA (1:500 in tissue, 1:100 in MN culture; monoclonal; Covance, RRID: AB_663239) served as a marker for cell replication and DNA repair. Primary antibodies were diluted in 2% NDS or NGS/PBS in 3% BSA containing 0.3% Triton X-100. Tissue specimens and MN-enriched cell cultures were incubated with primary antibody solution for 2 nights at 4°C. After washing in PBS, the samples were kept in the dark and incubated in the appropriate secondary antibody solution for 2 h. After a washing step, the cell nuclei were counterstained with 4′,6-Diamidino-2-phenylindole dihydrochloride (DAPI; Sigma-Aldrich) for 5 min, washed again with PBS and mounted with Fluoromount-G (SouthernBiotech, Birmingham, AL, USA).

For analyses, the samples were imaged as z-stacks using confocal laser scanning microscopy (LSM 710; Zeiss) with 40x and 63x immersion oil objectives for the tissue specimens and the MN cultures, respectively. For the cervical spinal cord specimens, the focus was set on the ventral horn, which was identified according to its typical morphology. For image processing, including adjustments of gamma settings, ZEN 2012 software (Zeiss) was used. Z-stack images of the ventral horn were overlaid as “maximum intensity projection”. For quantitative analysis of nuclear γH2AX foci in cultured astrocytes of the feeder layer, astrocytes were selected according to their size and morphology, and their nuclei were discriminated as the region of interest (ROI) by the DAPI signal. The extent of the γH2AX signal within the ROI was calculated as ‘integrated density per nucleus’ using ImageJ software [23] and is presented in arbitrary units (AU) ± SEM.

### Quantitative real-time PCR (qPCR)

Adult control and *hSOD1*^*G93A*^ transgenic mice were anesthesized with vaporized Isoflurane in a specialized chamber and sacrificed by decapitation. The spinal cord was micro-dissected and the extracted tissues were snap-frozen in liquid nitrogen. RNA was isolated according to standard protocols using QIAzol lysis reagent (Qiagen, Hilden, NW, Germany). The RNA solution was then treated with the TURBO DNA-*free*^™^ Kit (Invitrogen^™^/Thermo Fisher Scientific) to remove putative genomic DNA contamination. The RNA concentration and purity were determined by spectrophotometry (NanoDrop 2000c; Peqlab/VWR, Darmstadt, HE, Germany). Mean ratios of 2.02 ± 0.002 and 2.04 ± 0.004 for the 260 nm/280 nm and 260 nm/230 nm absorbance values, respectively, revealed the absence of protein and phenol contamination. The absence of DNA contamination and the RNA integrity, assessed by the RNA Integrity Score, were determined using the QIAxcel RNA QC Kit v2.0 (Qiagen) via the QIAxcel Advanced system (Qiagen). After dilution to 100 ng/μl, RNA was transcribed into cDNA utilizing the RevertAid First Strand cDNA Synthesis Kit (Thermo Fisher Scientific).

Real-time qPCR was performed with Brilliant II SYBR Green QPCR Master Mix (Agilent Technologies, Santa Clara, CA, USA). A final cDNA concentration of 25 ng per 20 μl of sample volume including 500 nM specific primers was used. The primer sequences were designed to exclusively recognize specific target sequences using the Primer-BLAST Online tool [24]. The primer specificity and predicted target size were confirmed by capillary electrophoresis, and the sequences were as follows:

*L1*_*orl*_ (GenBank: D84391.1; amplicon size: 131 bp):
5’- AGTCTGTACCACCTGGGAAC-3’; 5’- GGCAAGCTCTCTTACAGGGAA-3’
*L1*_*spa*_ (GenBank: AF016099.1; amplicon size: 123 bp):
5’- ATTGAAGCTCACGGCACATTTC-3’; 5’- GATGACCACTTATTCTGCATGTCTT-3’
*MusrsL1r* (GenBank: J02793.1; amplicon size: 133 bp):
5’- CCGCAAGCTGGAAGTTCATTAG-3’; 5’- AATGGTGGCAGTAGGAGCACA-3’
*Piwil1* (NM_021311.3; amplicon size: 93 bp):
5’-CACGACGATCAGGGAGTGACC-3’; 5’-TTCCAGTCAGCTCAGGTGTTC-3’
*Argonaute RISC catalytic subunit 2* (*Ago2*) (NM_153178.4; amplicon size: 100 bp):
5’-ACGCTCTGTGTCAATACCCG-3’; 5’-TCCTTCAGCGCTGTCATGTT-3’
*Ki67* (NM_001081117.2; amplicon size: 80 bp):
5’-TGGTCACCATCAAGCGGAG-3’; 5’-AATACTCCTTCCAAACAGGCAG-3’
*Glyceraldehyde-3-phosphate dehydrogenase* (*Gapdh*) ([25]; amplicon size: 164 bp):
5‘-GGAAGTGTCAGGGGAGGAGA-3‘; 5‘-GGCTACTTGGCGGTGTACAT-3‘.

For qPCR, the following parameters were applied: heating to 95°C (10 min), DNA denaturation at 95°C (15 s), annealing at 60°C (30 s) and product elongation at 72°C (30 s), with the last three steps being repeated for 40 cycles. At the termination of each run, a melt curve analysis was performed to evaluate the product specificity. Data were obtained using Rotor-Gene Q Series Software 2.3.1 (Qiagen). The relative transcript fold changes were assessed according to Pfaffl *et al*. [26] using the delta-delta threshold cycle (ΔΔC_t_). The values were normalized against the housekeeping gene *Gapdh*, which was co-amplified in the same run. Biological replicates were assessed as technical duplicates that included a ‘no-template control’ in each run. Relative transcript fold changes were assessed from the mean value of two duplicates.

### Subcellular protein fractionation and western blot analysis

Adult mice were sacrificed, the ventral cervical and thoracic spinal cords were micro-dissected, and proteins were isolated with respect to their subcellular localization using the Subcellular Protein Fractionation Kit for Tissues (Thermo Fisher Scientific), according to the manufacturer’s recommendations. The protein concentrations of the different subcellular fractions were analyzed by Bradford Assay using QuickStart^™^ Bradford Dye Reagent (Bio-Rad, Hercules, CA, USA). For the western blot analysis, 10 μg of a distinct subcellular protein fraction was subjected to standard SDS-PAGE and blotted onto a Hybond-P PVDF membrane (GE Healthcare, Little Chalfont, ENG, UK). To block non-specific epitopes, the membrane was incubated in 5% low-fat powdered milk (Roth) in TBS containing 0.1% Tween^®^20 for 1 h prior to the application of primary antibody solution. The following primary antibodies were used: rabbit anti-TDP-43 (1:2,500; polyclonal; Acris Antibodies, RRID: AB_615042), rabbit anti-NF-κB p65 (1:1,000; polyclonal; Santa Cruz Biotechnology, Dallas, TX, US, RRID: AB_632037) and mouse anti-NF-κB p65-active subunit (1:1,000; monoclonal; Merck Chemicals, RRID: AB_2178887). Primary antibodies were incubated in 2% low-fat powdered milk (Roth) in TBS containing 0.1% Tween^®^20 overnight at 4°C. After washing with TBS containing 0.1% Tween^®^20, the membranes were incubated with the appropriate HRP-conjugated secondary antibodies (1:5,000; Santa Cruz Biotechnology). After a washing step, bands were revealed by chemiluminescence using Immobilon Western HRP Substrate solutions (Merck Chemicals) and documented using the LAS3000 (Fujifilm, Düsseldorf, NW, Germany) with the corresponding software. The soluble and chromatin-bound nuclear fractions were quantified using ImageJ software [23]. TDP-43 protein levels were normalized to p65 levels, and all values were relativized to mean control values.

### Statistical analyses

All values are expressed as the mean ± SEM, with the exception of the transcript level data, which are presented as the geometric mean ± SEM. For multiple comparisons of normally distributed datasets, one-way ANOVA and two-way ANOVA followed by the *post hoc* Holm-Šídák test were performed according to the parameters addressed. For non-parametric single and multiple comparisons, the Mann-Whitney U and Kruskal-Wallis tests were applied, respectively. A *p* value of 0.05 was defined as the threshold for significance. Statistics were conducted with Sigma Plot 13.0 software systems (Systat).

## Results

### The *hSOD1*^*G93A*^ mutation does not increase spinal cord susceptibility towards DNA strand breaks

**Fig 1:**
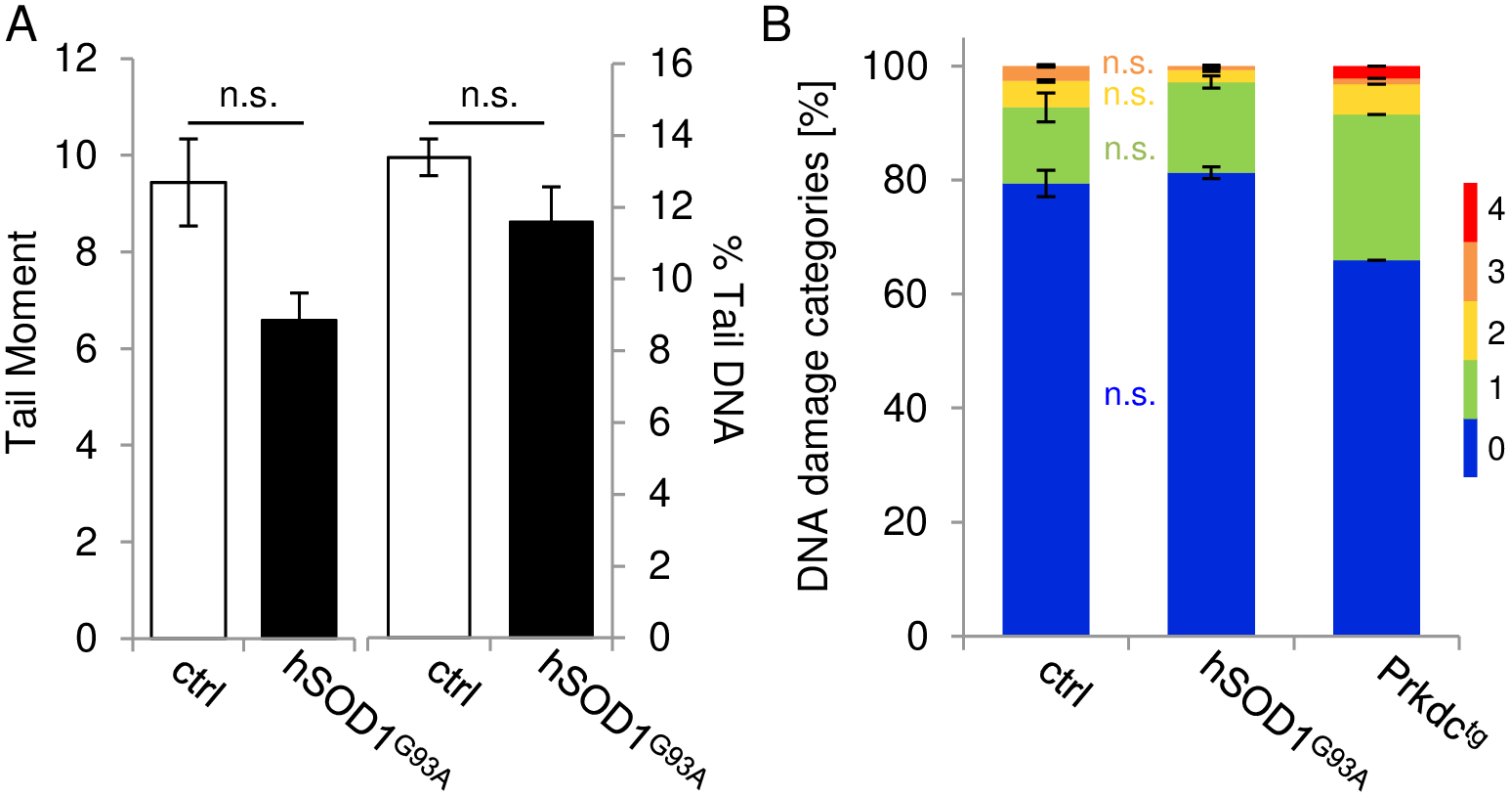
Assessment of DNA SSBs and DSBs in spinal cord tissue using the comet assay. **(A)** The extent of overall DNA damage, as expressed by Tail Moment (left bars) and % TailDNA (right bars) in cervical and thoracic spinal cord tissues, was similar between control (ctrl) and diseased *hSOD1*^*G93A*^ transgenic mice. **(B)** Specification of comet morphology by attribution to subclasses 0–4, according to increasing % TailDNA values (see Methods for details), revealed similar proportions of comets dedicated to each category between control and diseased *hSOD1*^*G93A*^ transgenic mice. A distinct pattern of DNA damage, however, was allocated to cortical cells from murine NOD.CB17-Prkdcscid/NCrHsd specimens displaying defective DNA repair (positive control; right bar), thus confirming assay sensitivity. n.s., not significant.

To assess the impact of the *hSOD1*^*G93A*^ mutation, implicating a toxic gain of SOD1 activity, on DNA integrity beyond base pair oxidation, the amount of deleterious DNA SSBs and DSBs was investigated in the spinal cord of control and severely diseased *hSOD1*^*G93A*^ mice. Cell nuclei from ventral parts of the cervical and thoracic spinal cord were subjected to the ‘comet assay’, in which voltage-based removal of nuclear DNA into a so-called ‘comet tail’ is considered to be directly proportional to the amount of DNA strand breaks. The extent of nuclear DNA distortion was determined as ‘Tail Moment’ and as the ‘percent of Tail DNA’ (% TailDNA) and classified according to the severity of DNA damage. The mean ‘Tail Moment’ did not differ between control (9.44 ± 0.98) and diseased (6.58 ± 0.51) *hSOD1*^*G93A*^ transgenic mice (Fig 1A, left bars; *p* = 0.429; control: n = 282 comets, *hSOD1*^*G93A*^: n = 425 comets, quantified from 3 animals per group). Similarly, the mean % TailDNA remained low and accounted for 13.39 ± 0.9% and 11.59 ± 0.57% in the control and diseased *hSOD1*^*G93A*^ transgenic groups, respectively, thereby exhibiting no obvious differences (Fig 1A, right bars; *p* = 0.778; control: n = 282 comets, *hSOD1*^*G93A*^: n = 425 comets, quantified from 3 animals per group). To assess gradual effects on DNA integrity and exclude a possible masking effect arising, e.g., from the mutual equilibration of very strong and weak DNA damage, the % TailDNA results were further classified into categories ranging from 0 to 4 according to the severity of DNA disintegration, as recently suggested by Collins [20] (Fig 1B). Both under control and disease conditions, comets dedicated to category 0 (0–20% TailDNA) represented the largest fraction, accounting for 79.38 ± 2.31% and 81.31 ± 1.01% of the isolated cells, respectively (*p* = 0.618). Accordingly, the mean values of % TailDNA were also allocated to this category. Of all comets analyzed, 13.38 ± 2.53% in the control specimens and 15.88 ± 1.09% in the *hSOD1*^*G93A*^ transgenic specimens were further attributed to category 1 of mild DNA damage and were thus not significantly different from each other (*p* = 0.520). Similarly, in categories 2 and 3, which collectively comprised a marginal portion within the comet-positive cell population (category 2: 4.64 ± 0.2% in the control group versus 2.09 ± 0.28% in the transgenic group, *p* = 0.512; category 3: 2.6 ± 0.2% in the control group versus 0.72 ± 0.18% in the transgenic group, *p* = 0.627), no differences were detected between control and ALS-like conditions. In either group, comets fulfilling category 4 criteria, indicating that almost the entire DNA content had shifted to the comet tail, were not present. The suitability of the ‘comet assay’ to categorize DNA damage in CNS tissue was confirmed by co-analyses of cortical neural cells derived from murine NOD.CB17-Prkdcscid/NCrHsd specimens exhibiting severe DNA repair deficits. The overall pattern of comets dedicated to the DNA damage categories in this reference tissue clearly differed from the control and *hSOD1*^*G93A*^ transgenic specimens (Fig 1B, right bar), as reflected by a reduced proportion of cells that were devoid of DNA damage concomitant with an increased portion of comets assigned to DNA damage categories 1–4. This finding demonstrates that the sensitivity of the ‘comet assay’ was sufficient to subtly detect and discriminate the demise of DNA.

Hence, using the ‘comet assay,’ which is sensitive to both DNA SSB and DSB when performed under alkaline conditions [27], we obtained no evidence for increased DNA damage under conditions of *hSOD1*^*G93A*^-related ALS-like pathology. Since the *hSOD1*^*G93A*^ mutation is present in all somatic cells and is likely to influence either cell type of the nervous system, comet samples were not selected for neurons. Indeed, although MNs are likely to display the strongest vulnerability, ALS-related pathomechanisms are not restricted to this cell entity [28].

### DNA damage response is not activated in motor neurons carrying the *hSOD1*^*G93A*^ mutation

**Fig 2:**
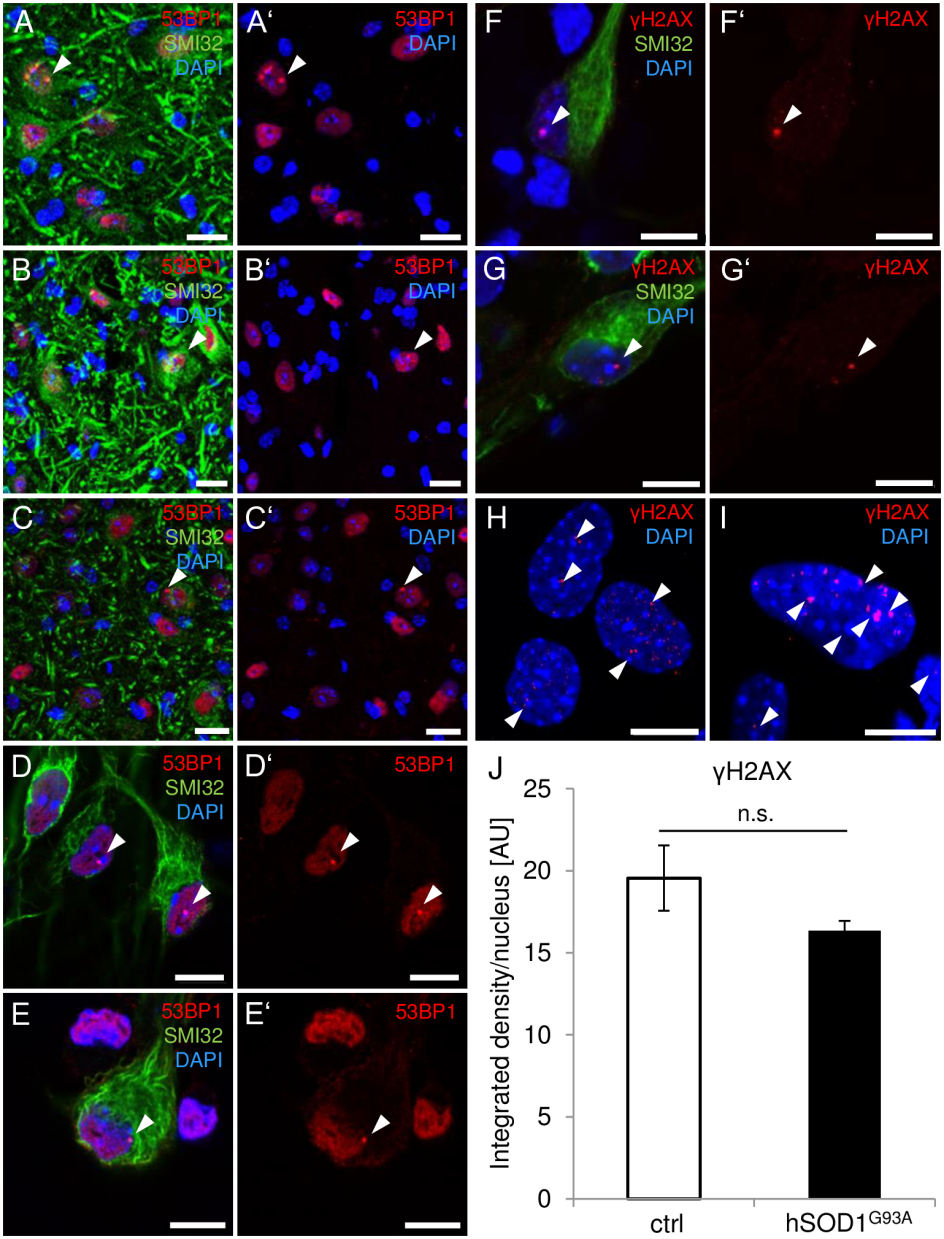
DSB events in spinal MNs *in vivo* and *in vitro*. **(A-C’)** Representative photomicrographs of 53BP1 immunoreactivity in the ventral cervical spinal cord of control **(A, A’)**, presymptomatic **(B, B’)** and diseased *hSOD1*^*G93A*^ transgenic mice **(C, C’)**. **(A-C’)** In MNs (green), 53BP1-positive foci (red) occurred at a very low frequency (arrowheads), independently of the underlying condition. **(D-E’)** Similarly, *in vitro* nuclear 53BP1 foci (arrowheads) were rarely detected both in control **(D, D’)** and *hSOD1*^*G93A*^ transgenic MNs **(E, E’)**. **(F-I)** Representative photomicrographs of γH2AX immunoreactivity *in vitro*. **(F-G’)** Nuclear γH2AX-positive foci (red; arrowheads) remained as single events both in MNs of non-transgenic **(F, F’)** and *hSOD1*^*G93A*^ donors **(G, G’)**. **(H, I)** *In vitro*, γH2AX-positive foci exhibited an apparently higher abundance in astrocytes both in control **(H)** and transgenic **(I)** cultures compared with MNs. **(J)** The amount of γH2AX foci in astrocytes *in vitro* did not differ significantly between control and transgenic astrocytes as quantified by means of integrated density per nucleus. Cell nuclei were counter-stained with DAPI (blue) to confirm the nuclear signal locations. n.s., not significant. Scale bars depict 20 μm (A-C’) and 10 μm (D-I).

Since it is not possible to discriminate between SSBs and DSBs in DNA by the comet procedure, at least in an alkaline milieu [27], we hypothesized that an equalizing shift in the proportion of either entity of DNA damage might mask DNA instability. To exclude this possibility and to explore the activation of DDR responses under *hSOD1*^*G93A*^-related ALS conditions, the appearance of nuclear 53BP1-positive foci was analyzed by immunofluorescence as a function of disease progression. The 53BP1 multi-domain marker operates as an early component of the DDR cascade by contributing to the processing of and signaling from sites of DNA damage [29]. Additionally, it serves as a robust marker specifically for DNA DSBs [29]. Therefore, 53BP1 activation was assessed in the cervical spinal cord tissue of control (n = 5), presymptomatic (n = 2) and severely diseased (n = 4) *hSOD1*^*G93A*^ mice, as well as in an α-MN-enriched cell culture system (Fig 2; n = 90 cells/preparation, 3 preparations). In general, the 53BP1 foci remained as single events both *in vivo* (Fig 2A–2C’) and *in vitro* (Fig 2D and 2E’), and they were not increased in *hSOD1*^*G93A*^ MNs. Similar results were obtained for γH2AX (Fig 2F–2I; n = 90 cells/preparation, 3 preparations), another well-established marker for DNA DSBs and DDR activation that co-localizes and interacts with several DDR mediators [30]. Again, γH2AX-positive foci remained discrete in cultured MNs derived from both control and transgenic mice (Fig 2F and 2G’). However, in replication-competent feeder layer astrocytes, there was a greater abundance of γH2AX-positive foci (Fig 2H and 2I). Quantification of nuclear γH2AX foci by assessment of the integrated density of γH2AX-positive signal frequencies per nucleus (Fig 2J) revealed 19.55 AU ± 2.00 AU and 16.35 AU ± 0.59 AU in the control (n = 600 cells/preparation, 3 preparations) and transgenic (n = 600 cells/preparation, 3 preparations) astrocyte cultures, respectively, and were thus similar among the groups (*p* = 0.216).

Taken together, we could not identify evidence for alterations in the ratio of SSBs:DSBs (Fig 2) in the *hSOD1*^*G93A*^ animal model of ALS, either under *in vivo* or *in vitro* conditions. Moreover, events indicating the relocation of DDR proteins such as 53BP1 to nuclear foci and the generation of γH2AX positive nuclear foci, e.g., to align broken DNA strands, were not increased.

### DNA repair is not increased in the spinal cord of *hSOD1*^*G93A*^ mutants

**Fig 3:**
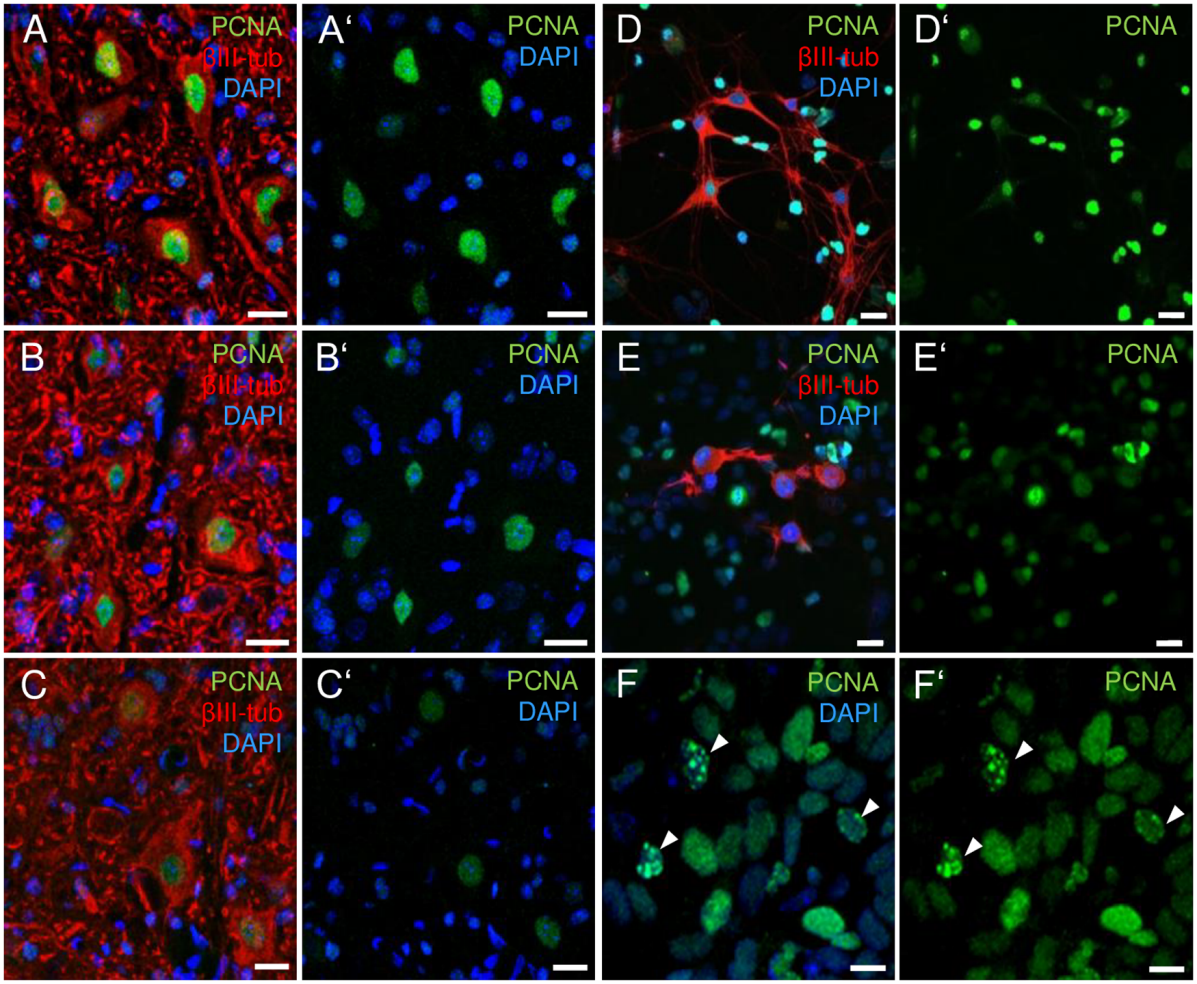
DNA damage repair in hSOD1^G93A^ transgenic mice. **(A-C’)** Representative photomicrographs of PCNA immunoreactivity in the ventral cervical spinal cord of control **(A, A’)**, presymptomatic **(B, B’)** and diseased *hSOD1*^*G93A*^ transgenic mice **(C, C’)**. In DAPI-counterstained MNs, identified by intense ßIII-tubulin staining (red) and morphological criteria, a nuclear PCNA signal (green) was present but did not show foci formation, neither in specimens of control nor *hSOD1*^*G93A*^ transgenic mice, independently of the presence of ALS-related symptoms. **(D-E’)** In addition, under *in vitro* conditions, PCNA-positive foci were missing in control **(D, D’)** as well as in transgenic neurons **(E, E’)**. **(F, F’)** However, PCNA-positive foci (arrowheads) were distinguishable in the brain tumor cells of a NOD.CB17-Prkdcscid/NCrHsd mouse displaying a high rate of cell replication. Scale bars are 20 μm (A-E’) and 10 μm (F, F’).

To further exclude that the proportion between SSBs and DSBs is influenced by molecular strategies that compensate for DNA damage, which are even beyond DDR mediator processes, we analyzed proliferating cell nuclear antigen (PCNA) as a potent marker for ongoing DNA damage repair [31, 32]. PCNA acts as a sliding clamp, which provides a platform for the recruitment of factors necessary for DNA replication and repair and is involved in the mediation of DNA damage tolerance pathways [32, 33]. In general, in immuno-stained cells, PCNA activation is indicated by the appearance of nuclear foci. Thus, in quiescent cells, as neurons that are devoid of S phase activity, positive PCNA foci are indicative of active DNA repair processes [32]. Regarding this feature, we analyzed the formation of nuclear PCNA-positive foci in the ventral cervical horn of control, presymptomatic and severely diseased *hSOD1*^*G93A*^ mice. These data were assessed to determine whether DNA repair in this transgenic strain was altered generically or whether it changed either before or after the onset of symptoms. In both control tissue (n = 6) and cervical spinal cords carrying the *hSOD1*^*G93A*^ mutation (presymptomatic: n = 3; diseased: n = 5), non-replicative neurons displayed a nuclear PCNA signal devoid of any foci, indicating that there were no active DNA repair processes (Fig 3A–3C’). Corresponding observations were obtained *in vitro* (Fig 3D and 3E’; n = 90 cells/preparation, 3 preparations). Similar transcript levels of the proliferation marker Ki67 under control and disease conditions (S2 Fig) further proved the lack of S phase induction. To confirm the capability of the PCNA antibody to generate foci-like signals, either in S phase or as a sign of DNA repair, human LN229 cell line-derived brain glioblastoma cells xenografted into NOD.CB17-Prkdcscid/NCrHsd mice were stained with an identical antibody directed against PCNA (Fig 3F and 3F’). Thus, highly replicative tumor cells showed strong nuclear PCNA foci. Consistent with the defect in the DDR apparatus in this mouse model, cells in tumor-free brain areas remained devoid of PCNA foci. Prkdc is a serine/threonine-protein kinase that is critically involved in DDR mechanisms such as DSB repair, the alignment of broken DNA ends and the prevention of chromosomal end fusion [34]. In light of these findings, it appears unlikely that MNs bearing the *hSOD1*^*G93A*^ mutation perform increased compensatory DNA repair processes, potentially masking an increase in DNA strand breaks.

### Transposable element activation is not elevated in *hSOD1*^*G93A*^ mutants

**Fig 4:**
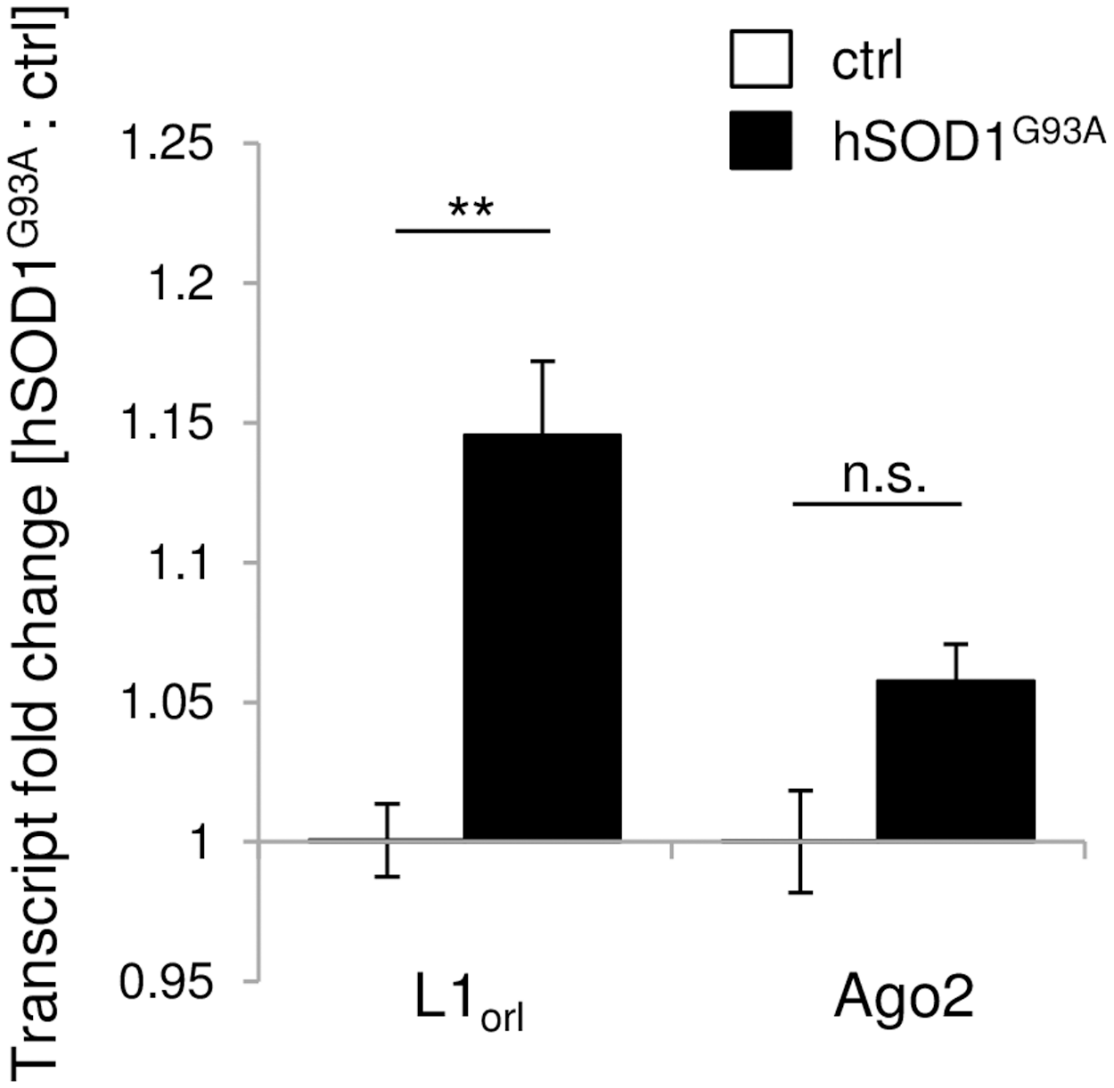
TE regulation in diseased *hSOD1*^*G93A*^ mutants *in vivo*. The relative levels of the TE *L1*_*orl*_ transcripts were significantly increased in the spinal cord of diseased *hSOD1*^*G93A*^ mice compared with controls. In contrast, spinal transcripts of *Ago2*, as part of the Ago2-RISC TE silencing complex, did not differ significantly between control and fALS-like conditions. **, *p* < 0.01. n.s., not significant.

In addition to metabolic DNA toxicity arising from the *hSOD1*^*G93A*^ mutation, endogenous cues might contribute to DNA instability. The integration of TEs into new genomic loci represents such an endogenous cue because it can cause DNA damage, e.g., DSBs [16]. Therefore, we investigated the spinal transcript levels of three murine retrotransposable long interspersed nuclear elements 1 (LINE1), L1_orl_, L1_spa_ and MusrsL1r, with LINE1 representatives encompassing the most frequent TE type in mammals [9], by qPCR. The transcription of TE-DNA into intermediate RNA templates is the first step in retrotransposition [9]. Among these three LINE1 elements, L1_orl_ displayed substantial levels, whereas transcripts of L1_spa_ and MusrsL1r remained below the sensitivity level of our SYBR Green-based qPCR system in both control and diseased *hSOD1*^*G93A*^ transgenic mice. The L1_orl_ transcript levels in the spinal cord of diseased *hSOD1*^*G93A*^ transgenic mice (Fig 4; 1.145 ± 0.027; n = 5) were significantly increased compared to control mice (Fig 4; 1.000 ± 0.013; n = 4; *p* = 0.008). In addition to TE transcript levels, we investigated the relative expression of *Ago2* and *Piwil1*. These two factors are part of two distinct TE silencing pathways that contribute to the degradation of TE transcripts. Ago2 forms a complex with RISC (RNA-induced silencing complex), whereas Piwil1 is part of TE transcript degradation via the piRNA pathway [35, 36]. *Piwil1* expression remained below the detection threshold in CNS tissue. Since we could detect *Piwil1* expression in testis as a positive control using the same detection system, we excluded a possible malfunction of the primer pair. In contrast, *Ago2* transcript levels were detectable in the spinal cord and were similar in control mice (Fig 4; 1.000 ± 0.018; n = 4) and diseased *hSOD1*^*G93A*^ transgenic mice (Fig 4; 1.057 ± 0.013; n = 5; *p* = 0.058). Thus, there was neither a compensatory increase nor a reduction in RISC-mediated TE silencing, suggesting no increased TE activation. Among all parameters analyzed, only the L1_orl_ transcript level was significantly increased. Therefore, we assume that elevated TE activation is unlikely to occur in the *hSOD1*^*G93A*^-dependent ALS-like pathology.

### TDP-43 mislocation is not a feature of *hSOD1*^*G93A*^ mutants

TDP-43 has recently been described to silence DNA toxic TE transcripts [10], and the loss of nuclear TDP-43 content might impair such a neutralizing function. Thus, we investigated this feature to validate further the exclusion of an increase in TE activation in a *hSOD1*^G93A^-dependent ALS-like pathology as a source of endogenous cues that potentially lead to DNA instability.

**Fig 5:**
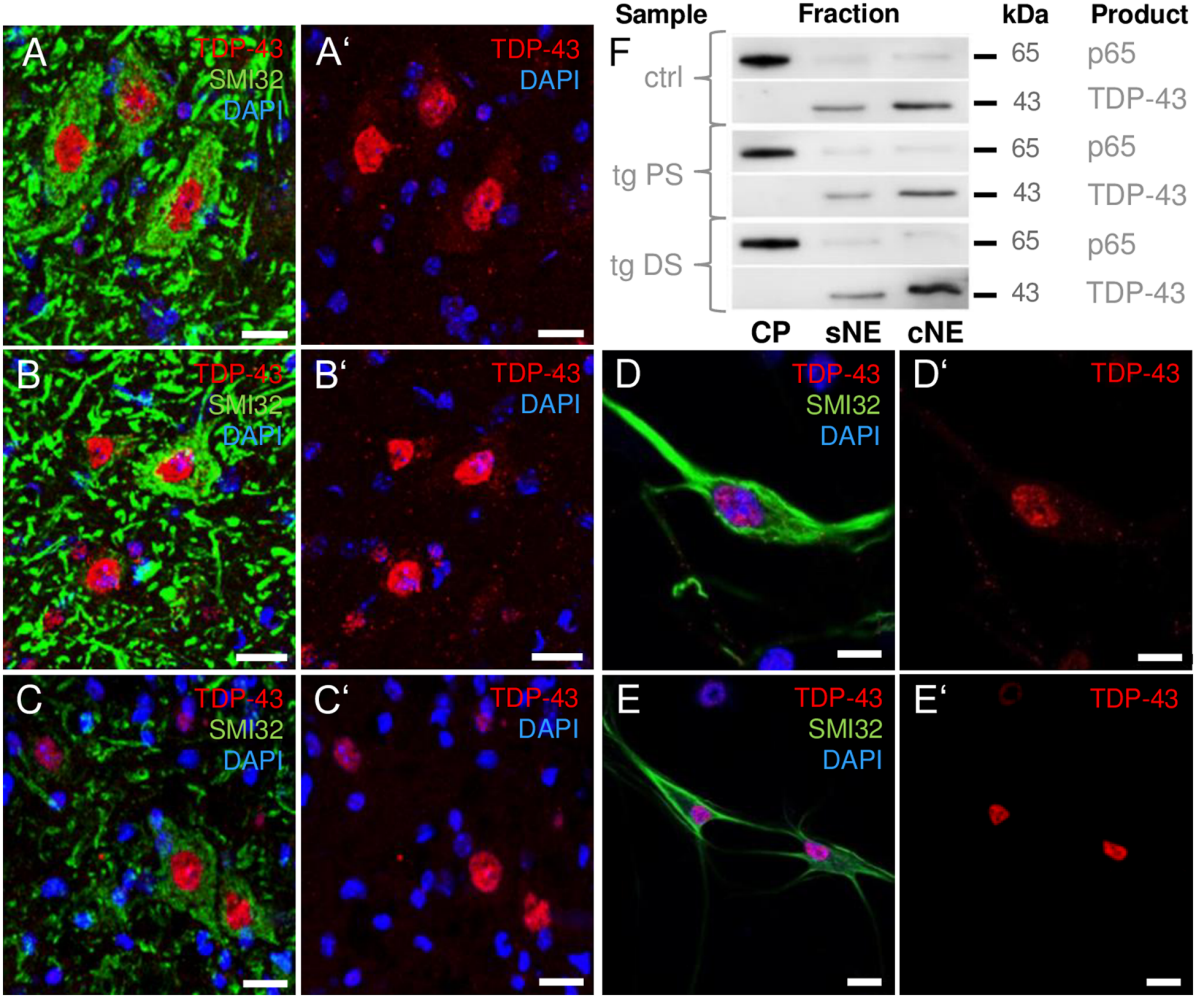
Subcellular TDP-43 distribution in MNs of mutant *hSOD1*^*G93A*^ mice *in vivo* and *in vitro*. (**A-C’**) Representative photomicrographs depicted in the ventral cervical spinal cord of control **(A, A’)**, presymptomatic **(B, B’)** and diseased **(C, C’)** *hSOD1*^*G93A*^ transgenic mice. In MNs (green), the cytoplasmic TDP-43 signal (red) did not exceed background levels, whereas the nuclear TDP-43 signal was strong in specimens of both control and *hSOD1*^*G93A*^ mice. Similar to *in vivo* conditions, cultured **(D-E’)** non-transgenic **(D-D’)** and transgenic **(E, E’)** MNs displayed a comparably low cytoplasmic TDP-43 signal, which was not increased in MNs carrying the *hSOD1*^*G93A*^ mutation. Nuclear TDP-43 location was verified by DAPI counterstaining (blue). **(F)** Representative western blot of TDP-43 in cytoplasmic (CP), soluble nuclear (sNE) and chromatin-bound nuclear (cNE) subcellular extracts derived from ventral cervical and thoracic spinal cords of control (ctrl), presymptomatic (PS) and diseased (DS) hSOD1^G93A^ mice. Under resting conditions, detection of cytoplasmic p65 (RelA) was utilized as a loading control and for purity validation of subcellular fractions. The cytoplasmic fraction was free of TDP-43, whereas TDP-43 was present in both nuclear extracts, with a higher abundance in the cNE than the sNE fraction. Scale bars depict 20 μm (A-C’) and 10 μm (D-E’).

At present, cytoplasmic mislocation of TDP-43 in *hSOD1*-related ALS pathologies is not well defined and remains controversial [37–40]. Therefore, the subcellular compartmentalization of TDP-43 in the *hSOD1*^*G93A*^ model was specified. Cervical spinal MNs of presymptomatic and diseased *hSOD1*^*G93A*^ mice as well as *hSOD1*^*G93A*^ transgenic MN-enriched cell cultures were analyzed for the presence or absence of cytoplasmic TDP-43 inclusions or the loss of nuclear TDP-43 content by immunofluorescence and fractionated immunoblotting (Fig 5). In general, SMI-32-positive α-MNs of the ventral cervical horn displayed a strong nuclear TDP-43 signal both in control (Fig 5A and 5A’, n = 4) and *hSOD1*^*G93A*^ transgenic animals (Fig 5B and 5C’; n = 6). Nuclear signal intensity remained stable irrespective of the underlying presymptomatic (Fig 5B and 5B’; n = 3) or severe (Fig 5C and 5C’; n = 3) disease phenotype. However, cytoplasmic TDP-43 signal was low to negligible in either condition (Fig 5A–5C’). For further consolidation of this observation, proteins from ventral cervical and thoracic spinal tissues were extracted, and the presence of TDP-43 was analyzed with respect to its subcellular localization. In the cytoplasmic extracts, TDP-43 was absent, whereas TDP-43 was strongly detected in the nuclear fraction both in control and *hSOD1*^*G93A*^ transgenic mice (Fig 5F and S1 Fig; n = 7 per group, non-pooled). We observed a stable nuclear TDP-43 level within the soluble nuclear fraction (control: 1.000 ± 0.483; presymptomatic *hSOD1*^*G93A*^: 0.804 ± 0.358; diseased *hSOD1*^*G93A*^: 1.095 ± 0.434; *p* = 0.925) and within the chromatin-bound nuclear extract (control: 1.000 ± 0.478; presymptomatic *hSOD1*^*G93A*^: 0.773 ± 0.304; diseased *hSOD1*^*G93A*^: 1.478 ± 0.604; *p* = 0.534) in *hSOD1*^*G93A*^ mice compared to control mice, independently of disease progression. According to its function in binding chromosomally integrated transactivation response element DNA, the presence of TDP-43 in the chromatin-bound nuclear fraction exceeded its soluble nuclear content (Fig 5F). The absence of cytoplasmic translocation or nuclear loss of TDP-43 was also shown in cultured MNs (Fig 5D and 5E’; n = 90 cells/preparation, 2 preparations). Low levels of cytoplasmic TDP-43 were detectable in some MNs in both *hSOD1*^*G93A*^ transgenic and non-transgenic cultures, but to a similar extent. Thus, there was no increase in cytoplasmic TDP-43 or loss of nuclear TDP-43 in MNs under *hSOD1*^*G93A*^-related ALS-like conditions compared with healthy controls, independent of the presence of symptoms.

## Discussion

The impact of DNA strand breaks and repair in non-replicative tissues and organs such as the nervous system and their importance in neurodegenerative disorders is not well understood. In particular, the relevance of severe DNA damage in multifactorial ALS pathogenesis is unclear. Distinct genetic requirements are prerequisite for DDR sensing and the repair of euchromatic and heterochromatic strand breaks. The DDR mediators 53BP1 and γH2AX are sensitive markers of DNA damage and part of the slow process of DSB DNA repair in G_0_/G_1_ phase cells, and PCNA is a potent element in the active tethering of DNA repair elements in non-replicative tissue [33, 41]. By addressing the DNA strand structure linked to these three DDR mediators, our data provide substantial evidence that, at least in a murine environment devoid of artificial stressors, the p.G93A mutation in the *hSOD1* gene is unlikely to increase cellular susceptibility to devastating DNA damage in terms of SSBs and DSBs. These phenomena occurred irrespective of the presence of a clinical phenotype.

Quantitative investigations of DNA strand breaks were initially based on single cell gel electrophoresis, termed the ‘comet assay’, and these data could be confirmed using DDR markers such as γH2AX. One advantage of γH2AX is its extraordinary sensitivity to unmask even very subtle alterations in genomic integrity at the single cell level, which makes it also applicable as a diagnostic tool in clinical settings [42]. One disadvantage of γH2AX is the frequently observed high background level of nuclear foci. However, such masking effects are commonly associated with DNA replication in dividing cells, but they occurred at rather low levels in our non-replicative neurons, which cannot transit through mitosis. Since the phosphorylation of H2AX occurs in parallel with other DDR substrates such as 53BP1, which also co-localizes at sites of activated DDR, the absence of either signal, as observed both *in vivo* and *in vitro*, again underscores the sensitivity and coherence of our analyses.

A protein that has recently been underestimated in terms of its genome restorative function is the DNA replication and repair marker PCNA, as evidenced, e.g., by its interaction with the DNA mismatch repair protein Msh2, the nucleotide excision repair protein XPG and the Rad6 pathway [31, 33, 43, 44]. Accordingly, DNA repair functions are not restricted to post-replication damage, but also cover stress-induced DNA demise [33, 45] and are executed by site-specific posttranslational modifications [33, 46]. Importantly, in quiescent cells PCNA transits from a soluble to an insoluble chromatin-bound state, appearing as foci, as soon as DNA repair activity is present [32]. With regard to the CNS environment, a homogenous missense mutation at p.Ser228Ile of the human *PCNA* locus has recently been identified to cause a neurodegenerative phenotype with clinical and molecular signatures of a DNA repair disorder, without affecting PCNA protein level and DNA replication [41]. Its application in postmitotic tissue together with DDR markers might thus be particularly useful due to its ability to detect active DNA repair processes apart from the execution of S-phase functions. Thus, in non-replicative neurons that are devoid of S-phase activity, the formation of PCNA-foci in terms of activation of the sliding clamp function in DNA restoration elements would be indicative of active DNA repair processes. In accordance with the results obtained using the ‘comet assay’ and DDR markers, PCNA foci formation did not increase in the *hSOD1*^*G93A*^-related ALS pathology. PCNA positive foci may manifest as a consequence of re-induced cell cycle activity, an event recently evidenced in neurodegenerative pathologies [47] including own observations in ALS-like pathology (will be published elsewhere). Proliferative activity may be distinguished from DNA damage by co-identification, e.g. of the Ki67 proliferation marker [48]. A corresponding analysis on the expression of Ki67 in the spinal cord of diseased *hSOD1*^*G93A*^ mice, using identical samples as for TE evaluation, proved unaltered Ki67 transcript levels, thus again underlining the absence of S phase induction. In conclusion, there was no sign for a compensatory elevation in DNA repair.

In contrast to our results, previous studies have described DNA fragmentation in the *hSOD1*^*G93A*^ mouse model as well as in *hSOD1*^*G93A*^-transfected SH-SY5Y human neuroblastoma-derived cells [49, 50]. In the lumbar spinal cord of *hSOD1*^*G93A*^ mice, Martin and colleagues discovered an increment of SSBs and DSBs with disease progression, as assessed using ssDNA antibody detection, PANT and TUNEL techniques with the latter known to be a commonly used cell death assay [49]. MNs of the lumbar spinal cord are particularly prone to undergo progressive cell death during disease manifestation [51], irrespective of the underlying genetic mutation. Hence, it cannot be excluded that this increase in DNA fragmentation observed by the authors occurred as a feature of an ongoing cell death process. In our study, the lumbar part of the spinal cord was excluded to avoid bias in the DNA damage analysis caused by rapid and massive neuronal loss. Similarly, with regard to a putative bias, the increased amount of DNA strand breaks detected in SH-SY5Y cells might be linked, at least to some extent, to cell cycle activity [50]. Further studies have also addressed the vulnerability of *hSOD1* mutated cells to DNA fragmentation, e.g., by applying artificial stressors. In support of our data, basal as well as X-ray-provoked levels of DNA damage were not increased in fibroblasts derived from *hSOD1*^*G93A*^-transgenic mice relative to controls [52]. Likewise, following exposure to artificially high ROS levels, DNA strand breaks in cultured MN-like NSC34 cells transfected with *hSOD1*^*G93A*^ were even reduced relative to naïve NSC34 cells, and higher levels of DNA damage in comparison to NSC34 overexpressing WT *hSOD1* were not increased above the levels detected in naïve NSC34 cells [7]. Two other studies found that *hSOD1*^*G93A*^ sensitizes mouse embryonic fibroblasts (MEFs) to oxidative stress, but only under DNA repair-deficient conditions [53, 54]. Experimentally, the *hSOD1*^*G93A*^ was expressed on an *Aprataxin*^*-/-*^ or a *Tdp1*^*-/-*^ (tyrosyl-DNA phosphodiesterase 1) background [53, 54]. Considering that both Aprataxin and Tdp1 are DNA repair enzymes, their absence might limit the cellular capability to compensate for *hSOD1*^*G93A*^-induced oxidative damage to DNA. Therefore, our data are in line with the finding that an overexpression of *hSOD1*^*G93A*^ requires additional stressors to evoke an increased susceptibility towards DNA strand breaks or DNA repair alterations.

Apart from such ROS-induced DNA toxicity, endogenous factors contribute to DNA disintegration. One such cue is represented by the integration of TEs into new genomic loci, which causes DNA damage such as DSBs [16]. There are several leads pointing to progressive de-repression and increased genomic integration of TE sequences during brain aging and neurodegeneration, including sALS pathology [9–12]. Moreover, although the associated molecular regulation is not yet understood, there is a preponderance of TE activation in neuronal tissue compared with other somatic tissues [15]. Hence, we analyzed different parameters that are indicative of elevated TE activation involving the *hSOD1*^G93A^ mutation in spinal cord tissue. Among them, we only identified a significant increase in L1_orl_ transcript levels. Likewise, in a group of 28 ALS patients, including a vast majority of sALS cases and several controls, Douville and colleagues detected an ALS-specific increase in the transcription of the retrotransposable human endogenous retrovirus (HERV)-K *pol* [12]. Moreover, the authors detected HERV-K reverse transcriptase in the cortical neurons of ALS patients, which co-localized with TDP-43 [12]. In light of such observations, it is tempting to assume that retrotransposons could play a role at least in sALS pathology, and further research is needed to define the importance of TE de-repression in both fALS and sALS. Our findings concerning TE activation in the *SOD1*^*G93A*^ model of fALS revealed a moderate increase in the transcript level of one of three LINE1 elements but did not clearly delineate whether TE activation occurred under this condition. Therefore, in addition to an unaltered expression level of *Ago2* as part of a TE silencing mechanism, we investigated a second, recently denoted TE transcript silencer, TDP-43 [10], which is closely associated with ALS pathology. TDP-43 is assumed to bind to TE-derived RNA transcripts, thus silencing mobile TE activity and protecting genomic integrity, e.g., by preventing DSB [9,10]. A reduced capability of TDP-43 in TE silencing has recently been associated with FTLD, and it has been found in mouse models displaying TDP-43 mutations [10]. Alterations in the subcellular location of TDP-43 might also impair the TDP-43 blocking function on genotoxic TEs. The presence and importance of cytotoxic cytoplasmic TDP-43 inclusions, specifically in *SOD1*-dependent ALS, are currently controversial [37–40], and alterations in the nuclear portion of TDP-43 are ill-defined. Some studies describe an absence of cytoplasmic TDP-43 but a reduction in nuclear TDP-43 content, which has been reported at least for fibroblasts derived from patients harboring an *hSOD1* mutation [39]. However, such a nuclear decrease seems to occur only in the co-existence of ubiquitinated cytoplasmic TDP-43 inclusions [55].

In the current study, nuclear TDP-43 remained stable in spinal MNs of *hSOD1*^*G93A*^ mice, and cytoplasmic TDP-43 did not appear. These results do not necessarily contradict the work of Sabatelli and colleagues because fibroblasts are fundamentally different from neurons and may react in a distinct way to the perturbations arising from a mutated *SOD1* gene. In support of our findings, Robertson *et al*. (2007) described the lack of TDP-43 pathology in several *SOD1* mouse models. However, cytoplasmic TDP-43 was present in two patients carrying a p.A4T and p.I113T mutation in the *hSOD1* gene [40]. Conversely, other studies including larger cohorts of ALS patients carrying an *hSOD1* mutation excluded the contribution of pathological TDP-43 to disease manifestation [37, 56]. Likewise, Mackenzie and colleagues (2007) analyzed spinal- or medulla-derived material from 111 ALS patients including 15 *hSOD1*-related fALS entities with the remaining 96 cases comprising sALS forms, cases of fALS caused by factors other than *hSOD1* mutations and ALS entities accompanied by dementia. Exclusively in patients carrying an *hSOD1* mutation, TDP-43-positive neuronal cytoplasmic inclusions were absent [37]. Similarly, Tan and coworkers described the lack of cytoplasmic TDP-43 in *hSOD1*-based fALS cases, at least when investigated in a small collection of CNS tissues [56]. Hence, the absence of cytoplasmic TDP-43 in murine *hSOD1*^*G93A*^ spinal MNs shown in the current study appears to resemble the situation in human *SOD1*-dependent fALS. Further studies that include large-scale genetic approaches with substantial explanatory power are needed to clarify this topic. In line with these observations, we did not obtain evidence for a *hSOD1*^G93A^-evoked malfunction of TDP-43, particularly in terms of its TE silencing function. Moreover, we found a preserved DNA integrity, devoid of additional severe DNA damage or compensatory DDR activation, in the *hSOD1*-dependent fALS-like pathology. Further studies are required to specify the role of TE activation in ALS patients and to define the integration of TE into new genomic loci, e.g., through the use of FISH techniques with human material.

## Conclusions

In summary, our data suggest that, in addition to the absence of TDP-43 mislocation and nuclear loss, *hSOD1*^*G93A*^ overexpression appears to have no impact on the ability of TDP-43 to silence transposable elements based on the absence of DNA damaging consequences. Furthermore, an increment in such genomic instability, i.e., in severe DNA damage such as DNA SSBs and DSBs, is unlikely to be evoked by *hSOD1*^*G93A*^ overexpression.

Characterization of other ALS-associated genes and DDR-related susceptibility factors such as senataxin (SETX) [57] and the newly discovered cyclinF [58] in their importance for DNA destabilization, as recently eventuated for *FUS* and *C9orf72* mutations [59, 60], might improve our understanding of ALS as a heterogeneous disorder.

## Supporting information

S1 Table Description of the clinical score used to assess the disease stage of the B6.Cg-Tg(SOD1*G93A)1Gur/J mice. (PDF)

S1 Fig The complete image of the western blot representative for p65 and TDP-43 protein levels as shown in Fig 5F. CP—cytoplasmic fraction; sNE—soluble nuclear extract; cNE—chromatin-bound nuclear extract; hSOD1^G93A^ PS—presymptomatic hSOD1^G93A^ mice; hSOD1^G93A^ DS—diseased hSOD1^G93A^ mice.
(PDF)

S2 Fig Proliferation marker Ki67 mRNA expression level. The relative levels of spinal Ki67 transcripts did not differ between control and fALS-like conditions. n.s., not significant.
(PDF)
