# Supplementary material for: DNA strand breaks and TDP-43 mislocation are absent in the murine hSOD1G93A model of amyotrophic lateral sclerosis in vivo and in vitro
Source: PLoS One. 2017 Aug 23;12(8):e0183684. doi: 10.1371/journal.pone.0183684 (PMC5568271; doi:10.1371/journal.pone.0183684)
Supplement: S1 Table — (PDF) [file pone.0183684.s001.pdf]

Supplementary Table 1.

Clinical score	Description
0	absence of symptoms evaluated by the ability of the animal to fully extent its hind limbs away from the lateral midline when suspended by its tail
1	a collapse of leg extension towards the lateral midline or a trembling of the hind limbs during tail suspension implying a starting hind limb paralysis
2	dragging along any part of the foot or curling the toes during walking
3	rigid paralysis along with the feet not being used for forward motion
4	humane endpoint is reached when the animal is not able to right itself within 30sec from either side
