# Supplementary figures and images for: DNA strand breaks and TDP-43 mislocation are absent in the murine hSOD1G93A model of amyotrophic lateral sclerosis in vivo and in vitro
Source: PLoS One. 2017 Aug 23;12(8):e0183684. doi: 10.1371/journal.pone.0183684 (PMC5568271; doi:10.1371/journal.pone.0183684)

Supplementary figure S1.

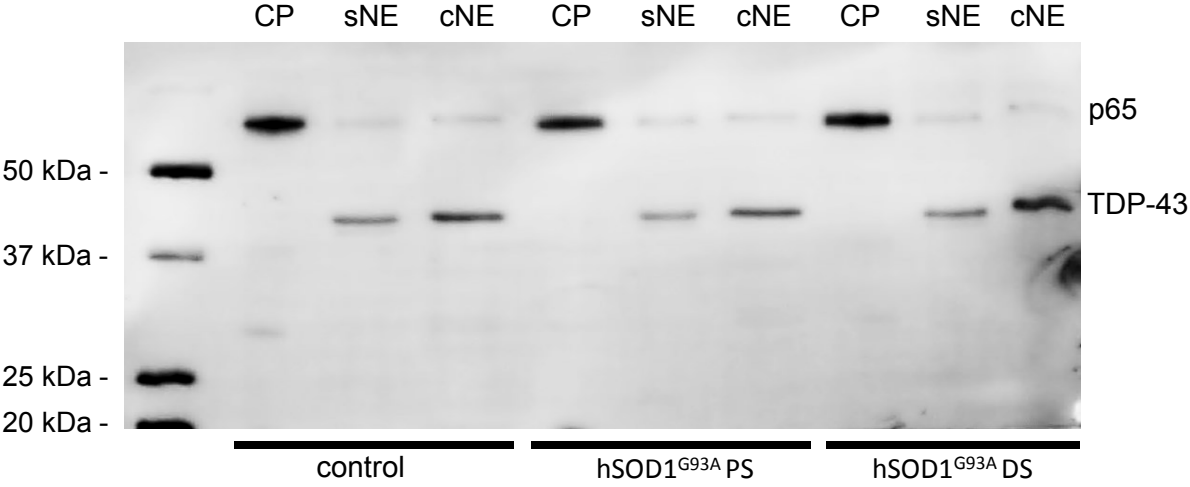

Supplement: S1 Fig — CP—cytoplasmic fraction; sNE—soluble nuclear extract; cNE—chromatin-bound nuclear extract; hSOD1G93A PS—presymptomatic hSOD1G93A mice; hSOD1G93A DS—diseased hSOD1G93A mice. (PDF) [file pone.0183684.s002.pdf]

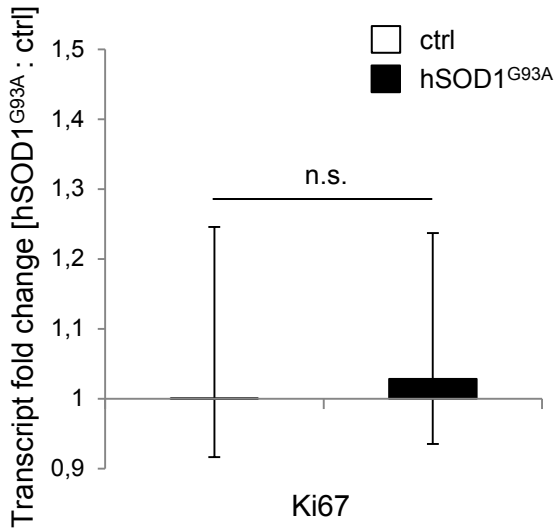

Supplement: S2 Fig — The relative levels of spinal Ki67 transcripts did not differ between control and fALS-like conditions. n.s., not significant. (PDF) [file pone.0183684.s003.pdf]
